# Anti-spike IgG Avidity Enhances Neutralization of Severe Acute Respiratory Syndrome Coronavirus 2: A Prospective Study of Primary Infections and Immunizations

**DOI:** 10.1093/infdis/jiag068

**Published:** 2026-03-05

**Authors:** Visa Nurmi, Lea Hedman, Katariina Vapalahti, Jussi Hepojoki, Hasan Uğurlu, Rommel Iheozor-Ejiofor, Chanice Knight, Kalle Saksela, Anu Kantele, Klaus Hedman, Olli Vapalahti

**Affiliations:** Department of Virology, Faculty of Medicine, University of Helsinki, Helsinki, Finland; Department of Virology, Faculty of Medicine, University of Helsinki, Helsinki, Finland; Department of Veterinary Biosciences, Faculty of Veterinary Medicine, University of Helsinki, Helsinki, Finland; Department of Medical and Clinical Genetics, Faculty of Medicine, University of Helsinki, Helsinki, Finland; Department of Virology, Faculty of Medicine, University of Helsinki, Helsinki, Finland; Institute of Veterinary Pathology, Vetsuisse Faculty, University of Zürich, Zürich, Switzerland; Department of Virology, Faculty of Medicine, University of Helsinki, Helsinki, Finland; Department of Molecular Biology and Genetics, Faculty of Sciences, Erzurum Technical University, Erzurum, Turkey; Department of Virology, Faculty of Medicine, University of Helsinki, Helsinki, Finland; Nuffield Department of Medicine, Peter Medawar Building for Pathogen Research, University of Oxford, Oxford, United Kingdom; Department of Virology, Faculty of Medicine, University of Helsinki, Helsinki, Finland; HUS Diagnostic Center, Clinical Microbiology, Helsinki University Hospital and University of Helsinki, Helsinki, Finland; Department of Infectious Diseases, Inflammation Centre, University of Helsinki and Helsinki University Hospital, Helsinki, Finland; Human Microbiome Research Unit, University of Helsinki, Helsinki, Finland; Meilahti Vaccine Research Center MeVac, University of Helsinki and Helsinki University Hospital, Helsinki, Finland; FIMAR, Finnish Multidisciplinary Center of Excellence in Antimicrobial Resistance Research, University of Helsinki, Helsinki, Finland; Department of Virology, Faculty of Medicine, University of Helsinki, Helsinki, Finland; HUS Diagnostic Center, Clinical Microbiology, Helsinki University Hospital and University of Helsinki, Helsinki, Finland; Department of Virology, Faculty of Medicine, University of Helsinki, Helsinki, Finland; Department of Veterinary Biosciences, Faculty of Veterinary Medicine, University of Helsinki, Helsinki, Finland; HUS Diagnostic Center, Clinical Microbiology, Helsinki University Hospital and University of Helsinki, Helsinki, Finland

**Keywords:** SARS-CoV-2, infectious diseases, immunology, neutralizing antibody, IgG avidity

## Abstract

**Background:**

Immune protection against coronavirus disease 2019 (COVID-19) relies, along with cellular immunity, on anti–severe acute respiratory syndrome coronavirus 2 (SARS-CoV-2) antibodies. We studied the effect of IgG avidity, the average antibody binding strength, on anti-SARS-CoV-2 neutralizing antibodies (nAbs), often considered the hallmark of effective immunity. Prior studies estimating the significance of avidity for nAb-mediated immunity have been complicated by the fact that not only the quality but also the quantity of antibodies impacts the results. Here we provide means for quantifying the impact of IgG avidity on neutralization, irrespective of antibody titer.

**Methods:**

We introduce for anti-SARS-CoV-2 spike protein (S) and nucleoprotein (N) antibodies, IgG avidity assays shown to be unaffected by the IgG concentration. Hospitalized (n = 14) and nonhospitalized (n = 14) COVID-19 patients and vaccinees (n = 20) of early 2020 were assayed for Wuhan S-IgM, S-IgA, S-IgG, and S-IgG avidity; Wuhan N-IgG and N-IgG avidity; and Wuhan, Beta, and Delta nAbs, to identify the factors contributing to neutralization efficiency.

**Results:**

N-IgG avidity was superior to S-avidity in pinpointing the time of SARS-CoV-2 primary infection. Both Wuhan nAb and Delta nAb correlated, expectedly, with Wuhan S-IgG level (*P* < .0001 each). Wuhan S-IgG avidity intensified homologous (Wuhan; *P* = .001) but not significantly heterologous (Delta; *P* = .053) neutralization. Accordingly, along with postinfection time, the average neutralization efficiency of S-IgG molecules increased while their concentration decreased. Quantitatively, doubling of Wuhan S-IgG avidity, at constant S-IgG quantity, augmented Wuhan neutralization 1.58- to 1.68-fold.

**Conclusions:**

Comprehensive serological profiles of early SARS-CoV-2 primary infections and immunizations provided a model showing that the antiviral neutralization potency is enhanced by anti-spike IgG avidity. The methodology presented is applicable widely beyond COVID-19.

## Teaser

Using primary infections and immunizations as model, each doubling of anti-spike IgG avidity enhanced SARS-CoV-2 neutralization 1.6-fold, independent of the corresponding IgG titer. When measuring IgG avidity, the IgG titer should be corrected for to avoid concentration-biased avidity results.

## Graphical Abstract

**Graphical Abstract:**
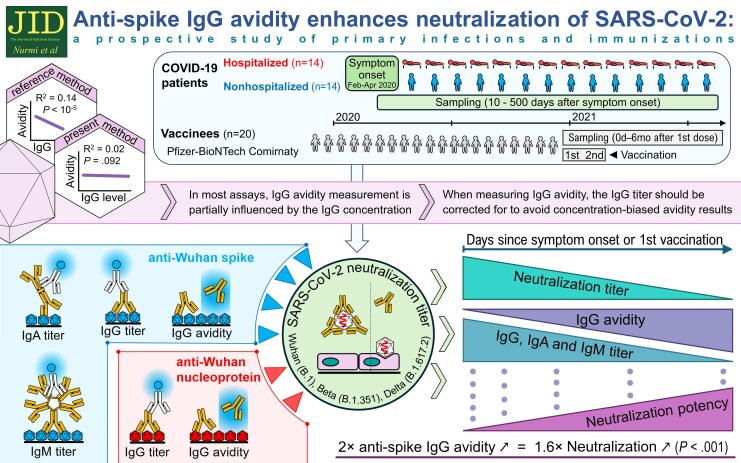

Severe acute respiratory syndrome coronavirus 2 (SARS-CoV-2) is the causative agent of the recent coronavirus disease 2019 (COVID-19) pandemic [1]. Together with cellular immunity, anti-SARS-CoV-2 antibodies, elicited by infection or vaccination, provide the main line of host defense against COVID-19. Higher anti-SARS-CoV-2 antibody titers have been observed during and following severe compared to mild COVID-19, reflecting stronger and wider immune activation [2]. After infection or immunization, protection from reinfections calls for recurrent antigen exposure, without which immunity against the continuously emerging variants remains short-lived [3, 4]. The avidity of anti-SARS-CoV-2 antibodies has been studied in detail [5–17]; however, the diversity of assays hampers in-depth assessments of the clinical impact of this immunodiagnostic parameter. Avidity has been assigned for a predictive marker of COVID-19 primary infection recovery [18], or as an indicator of long-term immunity [19]. While consistent maturation of spike protein avidity has been disclosed after vaccination [5, 6, 9, 13], cases of incomplete maturation postinfection also have been observed [6, 13]. Some reports even disclose that spike avidity declines postvaccination along with spike immunoglobulin G (IgG) and neutralizing antibody (nAb) decrease [7]. It also is largely unknown whether and to what extent antibody avidity influences neutralization of this virus, although such correlation has been proposed [5, 7–11]. Moreover, the efficacy of COVID-19 convalescent plasma therapy [20–26] may depend, in addition to quantity, on qualitative characteristics of the prophylactic antibodies.

In-depth knowledge of the anti-SARS-CoV-2 antibody response provides the basis for understanding the pathogenesis of and variant emergence with this virus. Early COVID-19 pandemic was pivotal in this regard, as much of the world's population first encountered an ancestral Wuhan-Hu-1–like strain (Wuhan), either via infection or vaccination, and the primary immune response can influence forthcoming infections and vaccinations via mechanisms such as original antigenic sin [27]. In the present work, we set to study the interdependencies of anti-SARS-CoV-2 spike protein (S) and nucleoprotein (N) antibody avidities with neutralizing antibody (nAb) potency against homologous and heterologous strains in consecutive samples following primary infections or immunizations (Figure 1*A*). We also aimed at quantifying the effect of IgG avidity on neutralization, independently of the corresponding IgG levels.

**Figure1.**
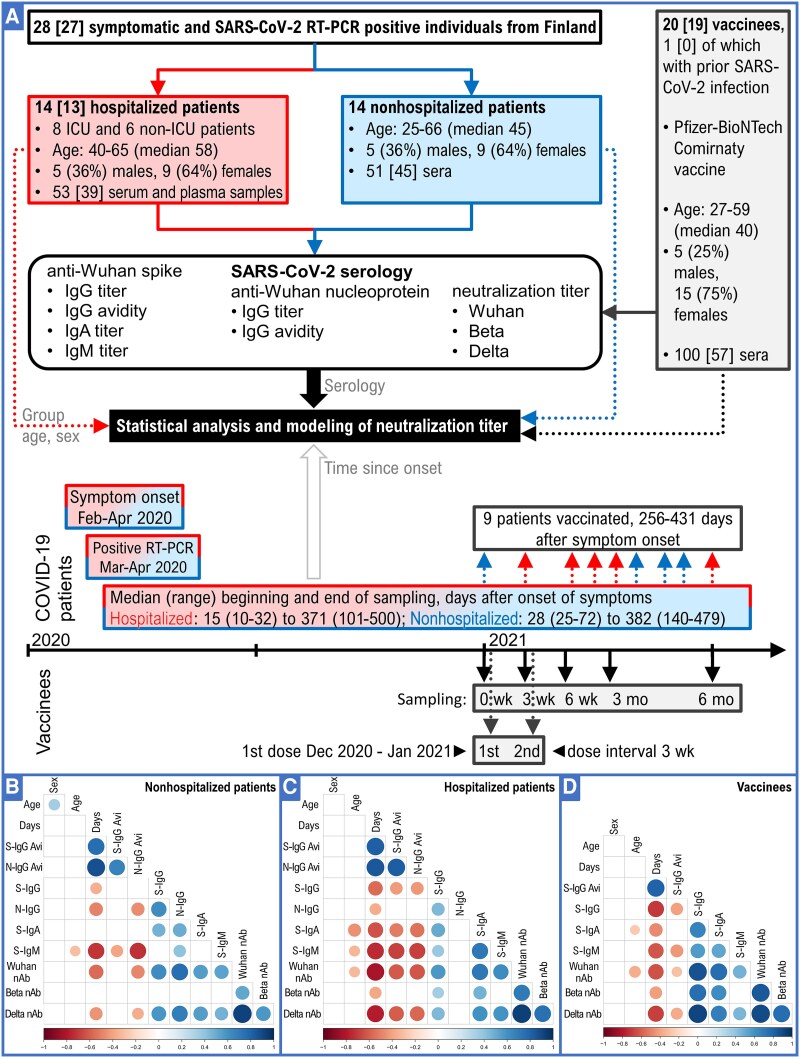
Experimental design. *A*, Anti-SARS-CoV-2 serological profiles of hospitalized and nonhospitalized COVID-19 patients and vaccinees were determined by assaying anti-Wuhan spike (S) IgG, S-IgG avidity, S-IgA and S-IgM; anti-Wuhan nucleoprotein (N) IgG and N-IgG avidity; and nAbs against Wuhan (B.1), Beta (B.1.351), and Delta (B.1.617.2) variants. The patients were followed up for 1 year (median) starting from shortly after primary SARS-CoV-2 infection in early 2020. Vaccinees were followed up for 6 months starting from administration of the first dose of Pfizer-BioNTech Comirnaty in December 2020–January 2021. Neutralization of SARS-CoV-2 was then analyzed based on the other serological results, time since symptom onset or vaccination, age, sex, and group (hospitalized, nonhospitalized, vaccinee). Numbers of individuals and samples included in multivariable models after data cleaning are shown inside square brackets. Correlations, in nonhospitalized (*B*) and hospitalized (*C*) COVID-19 patients and vaccinees (*D*), between S-IgG and N-IgG avidities; S-IgG, S-IgA, S-IgM, and N-IgG titers; and Wuhan, Beta, and Delta nAb titers. Dots show correlations with *P* < .05 and larger dot size indicates lower *P* value. Abbreviations: Avi, avidity; COVID-19, coronavirus disease 2019; ICU, intensive care unit; IgA, immunoglobulin A; IgG, immunoglobulin G; IgM, immunoglobulin M; nAb, neutralizing antibody; RT-PCR, reverse-transcription polymerase chain reaction; SARS-CoV-2, severe acute respiratory syndrome coronavirus 2.

## METHODS

### Subjects and Sera

#### COVID-19 Patients

Twenty-eight Finnish individuals with COVID-19 symptom onset in February–April 2020 tested SARS-CoV-2 positive by reverse-transcription polymerase chain reaction (PCR) at Helsinki University Hospital Diagnostic Center (Table 1). The clinical cohort comprised 2 groups. One included 53 sera or plasma samples from 14 patients (median, 3.5 [range, 2–6] samples per patient) hospitalized due to COVID-19 and sampled 10–500 days after onset of symptoms (median follow-up, 371 days). Median age was 58 (range, 40–65) years. Eight patients were treated at intensive care units during hospitalization. The other group comprised 51 sera from 14 nonhospitalized patients (median, 4 [range, 2–5] samples per patient) with milder COVID-19 presentation, sampled 25–479 days after onset (median follow-up, 382 days). Median age was 45 (range, 25–66) years. Both groups comprised 5 (36%) males and 9 (64%) females. Five hospitalized and 4 nonhospitalized patients were vaccinated during follow-up, 256–431 days after onset. The postvaccine samples were collected 13–149 days after vaccination.

**Table 1.** COVID-19 Patients and Vaccinees in the Present Study

Characteristic	Hospitalized COVID-19 Patients	Nonhospitalized COVID-19 Patients	Vaccinated Healthcare Workers
No. of individuals	14	14	20
No. of samples	53	51	100
Samples per individual, median (range)	3.5 (2–6)	4 (2–5)	5
Age at onset, y, median (range)	58 (40–65)	45 (25–66)	40 (27–59)
Male/female sex	5 (36%) / 9 (64%)	5 (36%) / 9 (64%)	5 (25%) / 15 (75%)
COVID-19 symptom onset	February–April 2020	February–April 2020	…
Infecting SARS-CoV-2 variant	Wuhan (B.1)	Wuhan (B.1)	…
SARS-CoV-2 vaccination	5 patients vaccinated in 2021, 310–421 d after symptom onset	4 patients vaccinated in 2021, 256–431 d after symptom onset	All vaccinated. First dose Dec 2020–Jan 2021, second dose 3 wk after first dose
Vaccine antigen	Wuhan variant spike	Wuhan variant spike	Wuhan variant spike
Sampling	10–500 d after symptom onset (median follow-up, 371 d)	25–479 d after symptom onset (median follow-up, 382 d)	0 d (prevaccine), 3 wk (pre–second dose), 6 wk, 3 mo, and 6 mo after first dose

Abbreviations: COVID-19, coronavirus disease 2019; SARS-CoV-2, severe acute respiratory syndrome coronavirus 2.

#### Vaccinees

The third group were 20 healthcare workers who received the first dose of Pfizer-BioNTech Comirnaty between December 2020 and January 2021, and the second dose 3 weeks after the first dose (Table 1). Median age was 40 (range, 27–59) years, and the group comprised 5 (25%) males and 15 (75%) females. The first sera were obtained prior to immunization, and follow-up samples were obtained 3 weeks (ie, before the second dose), 6 weeks, 3 months, and 6 months after the first dose. One individual had had SARS-CoV-2 infection prior to vaccination, as evidenced by the presence of anti-N antibodies.

### Anti-S and Anti-N IgG, IgA, and IgM Assays

Laboratory-derived anti-Wuhan S IgG, immunoglobulin A (IgA), and immunoglobulin M (IgM) and anti-Wuhan N IgG enzyme-linked immunosorbent assays (ELISAs) were used as described [28, 29]. Endpoint titers were acquired as described previously [30] and were normalized against calibrator sera (see Supplementary Methods for more details).

### Anti-S and Anti-N IgG Avidity

**Figure 2.**
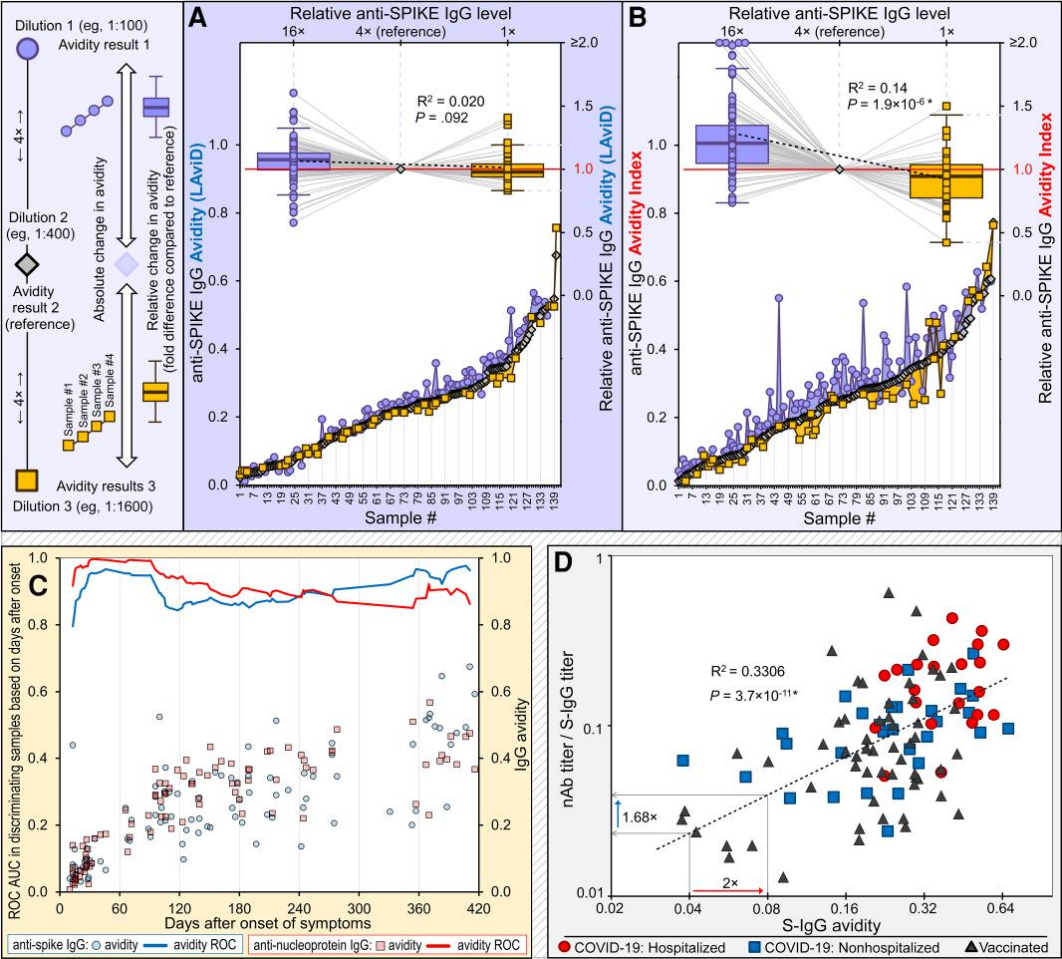
Effect of anti-Wuhan spike (S) IgG concentration on S-IgG avidity within linear range of the present avidity assay. Each sample (n = 140) was assayed at 3 sets of dilutions, 4-fold apart from each other. From the same raw data, avidities were obtained with the LAviD method (*A*; Nurmi et al [30]) used in the present study and, for comparison, with the popular “avidity index” approach (*B*; Hedman & Seppälä [32]). Change in avidity is show both in absolute avidity units and as relative within-sample variation. An ideal avidity result in not dependent on the IgG concentration it is measured at. *C*, Performance of S (n = 103) and anti-Wuhan nucleoprotein (N; n = 94) IgG avidity in discriminating samples based on time after symptom onset. ROC AUC values were calculated for each sampling timepoint (eg, AUC for discriminating samples collected within 60 days after onset from those collected later). N-IgG–negative follow-up samples were omitted from the N analysis. *D*, Wuhan (B.1) nAb titer to S-IgG titer ratio in relation to S-IgG avidity (n = 109). S-IgM has been shown to potently neutralize SARS-CoV-2, and samples with high (≥160) S-IgM titer were omitted to minimize confounding effect of S-IgM. Based on the regression model, doubling of S-IgG avidity increased nAb to S-IgG ratio 1.68-fold; ie, at constant S-IgG level the average neutralization potency of an S-IgG molecule increased 1.68-fold. Dashed black lines show linear regression. *Bonferroni-corrected *P* < .05. Abbreviations: AUC, area under the curve; COVID-19, coronavirus disease 2019; IgG, immunoglobulin G; nAb, neutralizing antibody; R^2^, adjusted *R*-squared value; ROC, receiver operating characteristic.

The S-IgG and N-IgG ELISAs were employed for measurement of the respective IgG avidities [26, 31]. Each sample was assayed with 2 series of dilutions, made at 4-fold steps (eg, series 1, 1:100 and 1:400; series 2, 1:400 and 1:1600). After antigen binding, series 1 was exposed to 4 M urea (in phosphate-buffered saline containing 0.05% Tween 20 [PBST]) 3 times 5 minutes each, and series 2 to PBST alone. Urea disrupts low-affinity antigen–antibody bonds whereas high-affinity bonds are more resistant. Both series were washed once with PBST before applying anti-human IgG conjugate. IgG avidity was calculated by the ratio Avidity = (IgG titer with urea, series 1) / (IgG titer without urea, series 2), using the LAviD method [30]. A ready-to-use spreadsheet for avidity calculation is provided in [30], and only requires input of dilution factors (series 1; series 2) and the respective absorbance results. Urea at 4 M had been found to best discriminate between samples collected within 1 month after onset of symptoms and those collected later. IgG avidity results in tests of many types have been shown to be influenced by the concentration of IgG [30, 32–35], and this effect was investigated first by assaying the samples at 3 sets of dilutions, 4-fold apart from each other (Figure 2*A* and 2*B*). For acquisition of the final avidity results, the S-IgG and N-IgG quantities in the present IgG avidity assay were further normalized by selecting the working dilution of each serum based on the initial screening—that is, the higher the IgG titer, the higher the dilution factor, resulting in absorbances of 0.5 ± 0.3, chosen to accommodate samples with low S-IgG or N-IgG concentration (Supplementary Figure 1). The LAviD calculation method [30] and avidity assay described here measure IgG avidity unbiased by IgG titer (Figure 2*A*, Supplementary Figure 1), a prerequisite for study of antiviral avidity as an independent factor of immunity.

### Neutralizing Antibodies

Neutralizing antibodies were titrated in a pseudovirus assay as described previously [36] using replication-defective lentiviruses pseudotyped with SARS-CoV-2 spike antigen of Wuhan (Wuhan-Hu-1 strain; B.1), Beta (B.1.351), or Delta (B.1.617.2) variants.

### Statistical Analyses

Factors in statistical analyses were sex at birth, age, time after symptom onset or first vaccine dose, group (hospitalized, nonhospitalized, or vaccinee), and serological markers measured in the present study (Figure 1*A*). Correlations were counted between all factors in each group and in the whole study population with R Hmisc package [37] and plotted using R corrplot package [38]. The mixed model (SAS Proc mixed procedure) was chosen as the most applicable method for modeling nAb titer (Wuhan or Delta), as the data were clustered, longitudinal, and unbalanced by timepoints. Linear regression models and receiver operating characteristic analyses were created with R 4.3.2 software (R Foundation for Statistical Computing, Vienna, Austria). The statistical methods are described further in the Supplementary Methods.

### Study Approval

The study was approved by the Ethics Committee of Helsinki University Hospital (HUS/853/2020; HUS/1238/2020). Informed consent was obtained from all subjects involved in the study.

## RESULTS

### Serology

**Figure 3.**
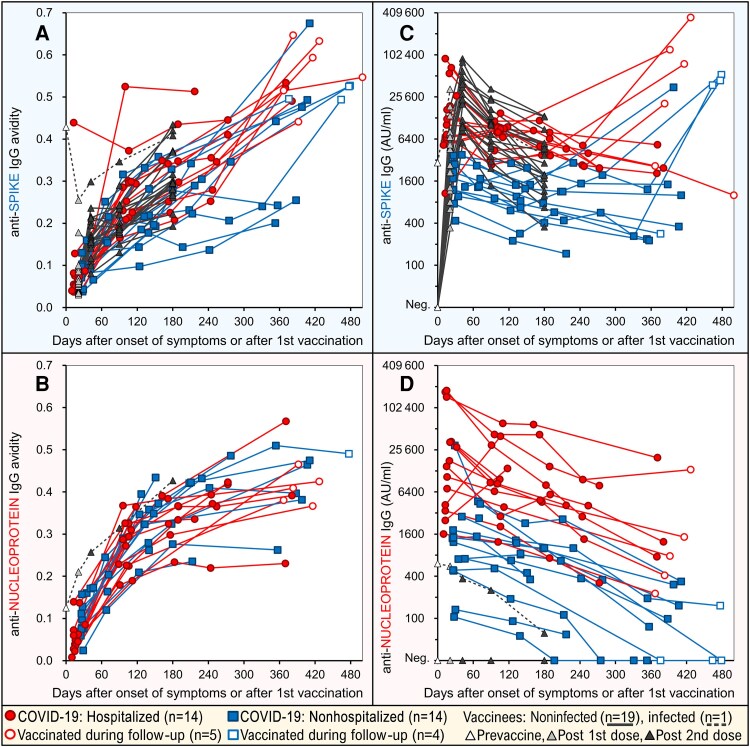
Anti-Wuhan spike (S) IgG avidity (*A*), anti-Wuhan nucleoprotein (N) IgG avidity (*B*), S-IgG titers (*C*), and N-IgG titers (*D*) of hospitalized and nonhospitalized COVID-19 patients and vaccinees in relation to time after onset (patients) or first vaccine dose (vaccinees). Abbreviations: COVID-19, coronavirus disease 2019; IgG, immunoglobulin G.

**Figure 4.**
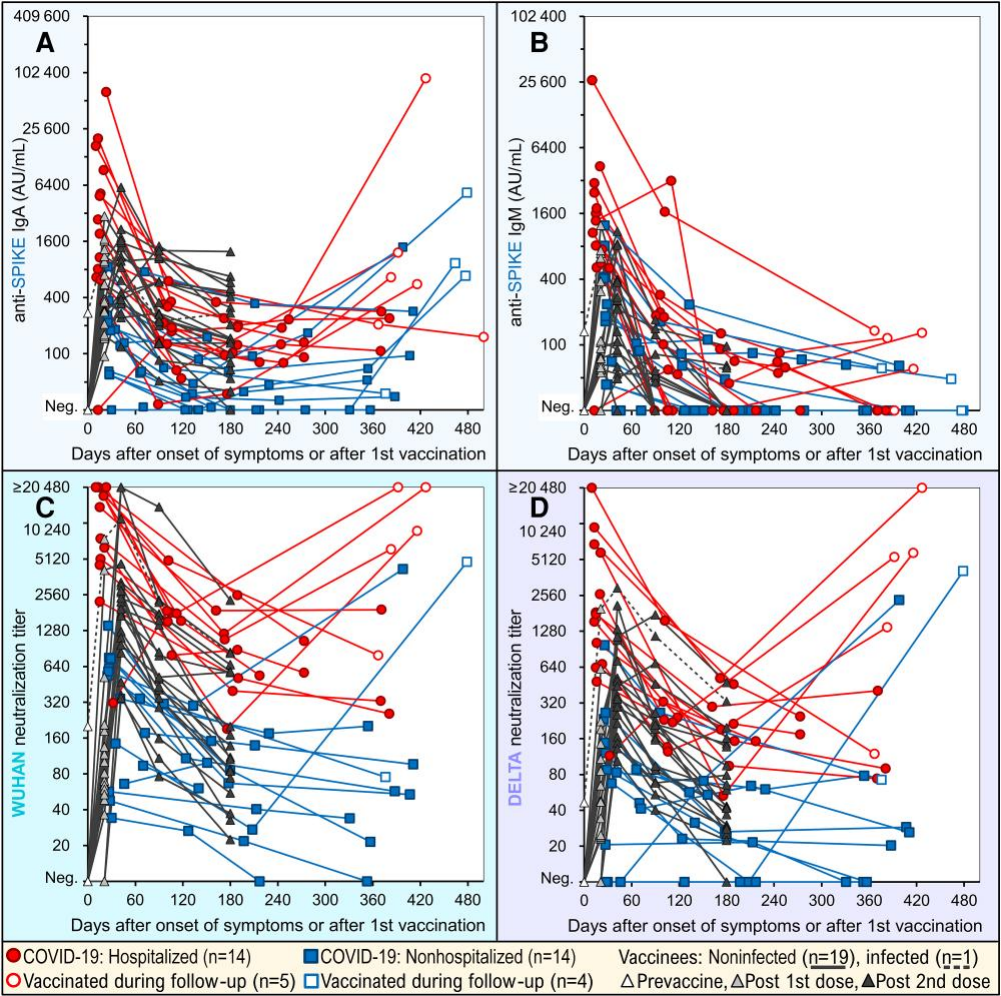
Anti-Wuhan spike (S) IgA titers (*A*), S-IgM titers (*B*), Wuhan (B.1) neutralization titers (*C*), and Delta (B.1.617.2) neutralization titers (*D*) of hospitalized and nonhospitalized COVID-19 patients and vaccinees in relation to time after onset (patients) or first vaccine dose (vaccinees). Abbreviations: COVID-19, coronavirus disease 2019; IgA, immunoglobulin A; IgM, immunoglobulin M.

In the present study (Figure 1*A*), hospitalized COVID-19 patients elicited on average higher antibody titers compared to nonhospitalized patients, for all antibody reactivities studied (S-IgG, S-IgA, S-IgM, N-IgG, nAb) and at all timepoints excluding samples taken after vaccine booster (Figures 3 and 4, Supplementary Figures 2 and 3). All 25 patients sampled within 5 weeks after onset tested S-IgM positive, while 3 nonhospitalized patients, sampled for the first time 41–72 days after onset, tested IgM negative (Figure 4*B*).

During follow-up, all antibody levels showed a decreasing trend in both patient groups (Figures 3 and 4, Supplementary Figures 2 and 3). All follow-up samples of hospitalized patients were seropositive for S-IgG, N-IgG, S-IgA, and Wuhan nAb, compared with 100% (14/14) S-IgG, 71% (10/14) N-IgG, 71% (10/14) S-IgA, and 86% (12/14) Wuhan nAb positivity in the latest samples of nonhospitalized patients (Figures 3 and 4). Due to variation in the sampling timepoints and lengths of follow-up, and limited sample sizes, accurate long-term seropositivities could not be obtained. Among previously infected subjects vaccinated during sampling, 7 of 9 showed significant (≥8-fold) increase in S-IgG, S-IgA, and nAb titers while 1 hospitalized and 1 nonhospitalized subject showed no additional response to vaccine (Figures 3*C* and 4).

Typically for primary infection, S and N IgG avidities were low at onset and matured during follow up (Figure 3*A* and 3*B*). Of note, S-avidity maturation lasted long, up to a year, apart from a few mildly infected and nonvaccinated individuals (Figure 3*A*). Four nonhospitalized subjects (29%) exhibited slow S-avidity maturation; their N-avidities as well as S-IgG titers, however, were unremarkable. Of these 4, a single subject was vaccinated on day 431, whereafter (day 464) her S-avidity showed enhanced maturation (from 0.24 to 0.49).

N-avidity matured uniformly between hospitalized and nonhospitalized patients but, compared with S-avidity, started to plateau 6 months after COVID-19 onset (Figure 3*B*). Altogether, N-avidity was superior to S-avidity in identifying recent primary infections (ie, discriminating between samples collected within versus beyond a given timepoint, from 1 month up to 8 months after onset; Figure 2*C*). S-avidity was better at later timepoints (eg, whether the sample was collected within or beyond a year after onset; Figure 2*C*). Additionally, all patients remained S-IgG positive, yet N-IgG is not influenced by S vaccines.

Among vaccinees lacking prior SARS-CoV-2 infection, antibody titers (S-IgG, S-IgA, nAb) resembled more those of the hospitalized COVID-19 patients than of the nonhospitalized patients (Figures 3 and 4, Supplementary Figures 2 and 3). All vaccinees were Wuhan nAb seropositive at 6 months (Figure 4*C*). S-avidity matured equally in vaccinees and hospitalized patients (Figure 3*A*, Supplementary Figure 3*A*). One individual infected before vaccination did show earlier S- and N-avidity maturation than noninfected vaccinees. Among both patients and vaccinees, the nAb titers correlated with the S-IgG, S-IgA, and S-IgM titers (Figure 1*B*–*D*). Wuhan nAb titers were, on median, 6.5-fold higher than Beta nAb titers and 3.4-fold higher than Delta nAb titers (Supplementary Figure 4).

### Modeling of Neutralizing Antibody Titers

To further investigate the factors influencing SARS-CoV-2 neutralization, 2 mixed multivariable models were selected based on evaluation using goodness of fit statistics (Akaike information criterion [AIC]; intraclass correlation coefficient [ICC]). The first model, referred to as the general model, included independent variables without any other interaction terms, except between sex and time. The second model, referred to as the interaction model, included additional significant interaction terms.

The general model, which focused on the Wuhan nAb titer as the outcome, included serological markers S-IgG, S-IgM, and S-IgG avidity, confounding factors sex, age, group (hospitalized, nonhospitalized or vaccinated), and days since onset of symptoms or since first vaccination (time) (Table 2). The interaction model used to compare effects of the serological markers between the 3 groups, was an expanded version of the general model with additional interactions of group with S-IgG, S-IgM, and S-IgG avidity (Supplementary Table 1). This interaction model was comparable to the general model in AIC (−260.1 and −251.2, respectively), ICC (0.534 and 0.513, respectively), and homoscedasticity (*P* = .730 and *P* = .676, respectively), and results for factors other than the group-interactions were parallel with the general model.

**Table 2.** General Model for Wuhan (B.1) Neutralization: Effect Estimates of the Mixed Multivariable Model Best Predicting Wuhan Neutralization Titer

Effect	Sex	Group	Estimate	Standard Error	*P* Value	Lower CI	Upper CI
Days	M	…	−0.553	0.087	<.0001^a^	−.725	−.381
	F	…	−0.441	0.073	<.0001^a^	−.587	−.296
Sex	M	…	0.510	0.022	<.0001^a^	.466	.553
	F	…	0.527	0.015	<.0001^a^	.496	.557
Age	…	…	−0.170	0.052	.002^a^	−.274	−.066
Group	…	Nonhospitalized	0.427	0.026	<.0001^a^	.376	.479
	…	Hospitalized	0.675	0.025	<.0001^a^	.624	.727
	…	Vaccinees	0.452	0.022	<.0001^a^	.408	.496
S-IgG titer	…	…	0.476	0.059	<.0001^a^	.358	.593
S-IgM titer	…	…	0.076	0.064	.235	−.050	.203
S-IgG avidity	…	…	0.271	0.077	.001^a^	.118	.423
Model evaluation criterion					Criterion value		
Akaike information criterion (the smaller the better)					−251.2		
Intraclass correlation coefficient (the higher the better)					0.513		
Homoscedasticity *P* value					0.676		
Measured vs predicted neutralization titer, adjusted *R*^2^					0.847		

Negative estimate value means inverse correlation, eg, neutralization titer decreases as days after onset of symptoms or first vaccination (days) increase, and vice versa. Abbreviations: CI, confidence interval; F, female; IgG, immunoglobulin G; IgM, immunoglobulin M; M, male; S, spike protein.

^a^ *P* < .05.

In the general model, an increase in S-IgG titer expectedly increased neutralization (*P* < .0001) as did also younger age (*P* = .002) (Table 2). Interestingly, increase in S-IgG avidity per se increased neutralization significantly (*P* = .001; Table 2), that is, maturation of avidity increased neutralization potency per unit of IgG. This was also supported by ratio of nAb to S-IgG correlating with S-avidity in the corresponding linear regression model (*R*^2^ = 0.33; *P* = 3.7 × 10^−11^; Figure 2*D*). S-IgM titer was not a significant predictor of neutralization in general (*P* = .235; Table 2), although it appeared to impact the neutralization ability of the sera collected from the nonhospitalized patients (*P* = .044; Supplementary Table 1). In the interaction model, decrease of nAb titer appeared faster for males than for females (Supplementary Table 1; type 3 effect *P* = .002). While N-IgG and N-avidity correlated with S-IgG and S-avidity, respectively (Figure 1*B* and 1*C*), a model with N-IgG levels and avidity turned out inferior in predicting nAb titers, as compared to S-IgG and S-avidity (Table 2).

The same mixed models fitted against Delta nAb yielded results parallel to those obtained for Wuhan nAb, yet predictive power and overall statistical significance were lower for heterologous neutralization (general model: AIC = −212.9, ICC = 0.447, homoscedasticity *P* = .202; and interaction model: AIC = −235, ICC = 0.413, homoscedasticity *P* = .047). Wuhan S-IgG was a statistically significant predictor of heterologous neutralization in the general model (*P* < .0001; Table 3) and Wuhan S-IgM in the interaction model (Supplementary Table 2) within hospitalized patients (*P* = .049) and vaccinees (*P* = .041). Effect of Wuhan S-IgG avidity for heterologous nAb levels against the Delta variant did not quite reach statistical significance in general (*P* = .053; Table 3) but was significant for nonhospitalized patients (*P* = .002; Supplementary Table 2), suggesting a putative effect.

**Table 3.** General Model for Delta Variant (B.1.617.2) Neutralization: Effect Estimates of a Mixed Multivariable Model Predicting Delta Neutralization Titer

Effect	Sex	Group	Estimate	Standard Error	*P* Value	Lower CI	Upper CI
Days	M	…	−0.396	0.102	<.0001^a^	−.509	−.194
	F	…	−0.339	0.086	<.0001^a^	−.509	−.169
Sex	M	…	0.337	0.023	<.0001^a^	.290	.385
	F	…	0.353	0.016	<.0001^a^	.320	.386
Age	…	…	−0.064	0.056	.263	−.177	.050
Group	…	Nonhospitalized	0.269	0.028	<.0001^a^	.212	.325
	…	Hospitalized	0.461	0.028	<.0001^a^	.406	.517
	…	Vaccinees	0.306	0.024	<.0001^a^	.258	.354
S-IgG titer	…	…	0.340	0.068	<.0001^a^	.204	.476
S-IgM titer	…	…	0.125	0.074	.094	−.022	.272
S-IgG avidity	…	…	0.175	0.089	.053	−.002	.352
Model evaluation criterion					Criterion value		
Akaike information criterion (the smaller the better)					−212.9		
Intraclass correlation coefficient (the higher the better)					0.447		
Homoscedasticity *P* value					0.202		
Measured vs predicted neutralization titer, adjusted *R*^2^					0.747		

For comparison, the same model was used as with Wuhan neutralization. Negative estimate value means inverse correlation, eg, neutralization titer decreases as days after onset of symptoms or first vaccination (days) increase, and vice versa.

Abbreviations: CI, confidence interval; F, female; IgG, immunoglobulin G; IgM, immunoglobulin M; M, male; S, spike protein.

^a^ *P* < .05.

In the models, estimates for anti-Wuhan S-avidity–derived enhancement of neutralization did not significantly differ between Wuhan and Delta variants (*P* = .54; Supplementary Table 3, Supplementary Figure 5). Albeit analyzed, the modeling approach did not yield feasible results with the Beta variant due to few samples demonstrating Beta neutralization beyond threshold. No model was able to accurately predict nAb titer at individual level (Supplementary Figure 6).

In the general mixed model (Table 2) and at constant S-IgG quantity, doubling of Wuhan S-avidity increased neutralization of the Wuhan variant 1.58-fold (Supplementary Figure 5A), and no difference in this effect was observed between the 3 groups (*P* = .144 to .803; Supplementary Table 3). In the linear regression model between the ratio of nAb to S-IgG, and S-IgG avidity (Figure 2*D*), the doubling of Wuhan S-avidity at constant S-IgG quantity increased Wuhan variant neutralization 1.68-fold. Although the data in these 2 approaches were not identical and the regression model did not account for the effect of time, it is still noteworthy that the 2 approaches yielded similar estimates for quantitative effect of avidity on neutralization, supporting validity of the models.

IgM responses among vaccinees were weaker than among patients. On average (as measured by geometric mean ratio), the vaccinees showed 3.0-fold higher S-IgG to S-IgM ratios than the patients (Supplementary Figure 3*J*). Accordingly, in the interaction models, Wuhan S-IgG was a significantly stronger predictor of both homologous and heterologous neutralization for vaccinees than for patients (*P* ≤ .004 for Wuhan nAb; *P* ≤ .014 for Delta nAb; Supplementary Table 3, Supplementary Figure 5*C* and 5*D*); and inversely Wuhan S-IgM was for patients than for vaccinees (*P* ≤ .021 for Wuhan nAb; *P* ≤ .006 for Delta nAb; Supplementary Table 3, Supplementary Figure 5*E* and 5*F*).

## DISCUSSION

In SARS-CoV-2 primary infection, IgG avidity toward the nucleoprotein matured equally among hospitalized and nonhospitalized patients, whereas toward the spike protein a proportion (29%) of nonhospitalized patients showed slower maturation. N-avidity was superior in diagnosing a primary infection and for estimation of the time since contagion, as implied earlier [6], and can also distinguish infection from S-based vaccination. Serology could thus complement clinical diagnosis of emerging respiratory pathogens, especially if PCR-based tests are in short supply. However, beyond half a year after infection, some of the nonhospitalized individuals lost their N-seropositivity, unlike any of the hospitalized patients. S-avidity was also better in identifying samples collected beyond 8 months after onset due to lengthier duration of avidity maturation.

The tardy maturation of S-avidity in some of the clinically mild patients (n = 4) was accompanied by normal N-avidity maturation and was not due to low S-IgG titer, and rapid S-avidity maturation was observed postvaccination. This suggests that low S-avidity after mild infection as such is not a marker of immune defect. On the other hand, high IgG avidity after severe infection or repeated exposure, for example, in vaccination series, could be a hallmark of protection as S-avidity was shown to correlate with neutralization capacity of SARS-CoV-2 Wuhan and Delta variants. S-avidity maturation, or lack of it, could help determine optimal vaccination intervals or recognize immunocompromised individuals who require an additional vaccine booster. Several reports have indeed advocated the utility of IgG avidity in study of immune protection and epidemiology of SARS-CoV-2 [6, 39, 40].

Higher S-IgG avidities have been reported in vaccinees than in patients [6, 12, 13] or in severe compared to mild clinical pictures [8, 41], at comparable sampling times postinfection or vaccination. In those studies, higher avidities were observed in the groups that also showed higher S-IgG titers. In the present work, some patients with mild symptoms showed particularly slow avidity maturation yet accounted for only 29% of patients, unlike 88% in another study [13]. Here, vaccinees and patients did not differ in terms of avidity. Correlation between high avidity and high S-IgG titer was not observed either, although when avidities were recalculated using the popular avidity index approach [32] and without normalization for S-IgG level, higher S-IgG concentrations showed bias toward higher avidities (Figure 2*B*).

IgG avidity assays of many types can indeed be influenced by the concentration of the corresponding IgG [30, 32–35], and many studies have investigated anti-SARS-CoV-2 IgG avidity without correcting for the IgG titer [5, 6, 8, 10, 13, 15, 16]. This dependence of antibody avidity (quality) on titer (quantity) may result in biased avidity results or in misleading association between, for example, neutralization and IgG avidity, if the IgG titer is not investigated and corrected for as a covariate [9, 14, 17, 30]. Since clinical avidity diagnosis often is based on tertiary classification (low, intermediate, or high avidity) [42], even relatively small avidity bias could result in misclassification and consequently change viral diagnosis or decision for starting antiviral medication. Concentration-corrected IgG avidity should thus be the clinical norm. The diversity of avidity assays is also extensive, and their results are expressed as arbitrary units, further hampering data comparison and emphasizing the importance of methods description and assay validation. Use of international avidity standards could improve harmonization and concordance between laboratories.

Neutralizing antibodies are often considered the gold standard of humoral protection, also with COVID-19. As neutralization assays tend to be laborious, other correlates of seroprotection have been sought. With SARS-CoV-2, the strongest correlate of neutralization, here and in the literature, was S-IgG titer measured in standard ELISA. S-IgM also correlated with neutralization to some extent; however, antibody quantity (S-IgG and S-IgM) did not fully explain the nAb titer. Additionally, Wuhan S-IgG avidity maturation affected neutralization of SARS-CoV-2 Wuhan variant independent of IgG titer and using an avidity assay shown to be unbiased by the level of corresponding IgG. Indeed, the average neutralization potency of an S-IgG molecule increased while their quantity decreased postinfection. Doubling of Wuhan S-avidity increased Wuhan nAb titer considerably, 1.58- to 1.68-fold in the 2 statistical models used. To the authors' knowledge, this is the first quantitative assessment of the extent by which IgG avidity contributes to neutralization of SARS-CoV-2. Corresponding estimates acquired via other avidity assays, for example, with another urea concentration, may differ numerically from the one presented here. The gold-standard live-virus neutralization test may also be preferred over the pseudovirus assay used in the present study, although such methods in general correlate well with each other [43]. While S-IgG, S-IgG avidity, and S-IgM did all contribute to neutralization, these parameters alone could not accurately predict nAb titer at an individual level. Correlation between S-IgG avidity and neutralization of SARS-CoV-2 has been reported previously [5, 7–11], however, in most instances lacking evidence on whether the avidity (quality of IgG) then is unbiased by the respective IgG quantity.

High IgG avidity against ancestral SARS-CoV-2 has been reported to increase cross-neutralization of variants of concern [5, 9, 11]. In the present study, the same trend was seen. However, the sample size of 48 individuals was a limiting factor, especially regarding heterologous neutralization, and did not allow the corrections for multiple comparisons in multivariable models and pairwise correlations. Here, Wuhan S-avidity increased cross-neutralization of Delta variant less than of homologous Wuhan neutralization, plausibly as the ability to evade preexisting immunity is crucial for each new SARS-CoV-2 variant. Since affinity maturation predominantly occurs following primary infection, the individuals studied here were sampled early and hence became immunized only with ancestral SARS-CoV-2. This was beneficial in eliminating immunological noise of reinfections, which are typically numerous and often undocumented. Yet, the consequent number of cross-neutralization positive individuals was not sufficient for statistical analysis of the Beta variant, let alone for the more diverse contemporary Omicron variants. Use of IgG avidity could improve estimates of cross-variant immunity and help understand population immunity and need for, eg, variant-specific booster vaccines. However, original antigenic sin may also hamper immune adaptation to viral evolution and result in unexpectedly low efficacy of said variant-specific boosters [44], in which case additional doses could be required. Optimal vaccination strategies of today are thus likely to depend also on knowledge of the prior immune response.

## CONCLUSIONS

We have here introduced IgG avidity assays based on SARS-CoV-2 S and N antigens for measurement of IgG avidity unbiased by concentration of the corresponding IgG, thereby allowing for accurate investigation of the role of antibody avidity in neutralization-mediated protection against COVID-19. IgG concentration–corrected avidity should be used, in general and beyond SARS-CoV-2, for improved accuracy. The strength of anti-S IgG avidity correlated with the neutralization potency, independent of IgG titer and time since primary infection or vaccination. We moreover provided methods for quantifying this effect: When comparing the same S-IgG quantities, doubling of their Wuhan S-avidity augmented Wuhan neutralization titer 1.58- to 1.68-fold. These approaches may have general utility beyond SARS-CoV-2 in diagnosis of primary infection and in assessment of time since contagion, neutralization potency, and vaccine protection.

## Supplementary Material

jiag068_Supplementary_Data

## Supplementary Data

Supplementary materials are available at *The Journal of Infectious Diseases* online (http://jid.oxfordjournals.org/). Supplementary materials consist of data provided by the author that are published to benefit the reader. The posted materials are not copyedited. The contents of all supplementary data are the sole responsibility of the authors. Questions or messages regarding errors should be addressed to the author.
