# Supplementary material for: Anti-spike IgG Avidity Enhances Neutralization of Severe Acute Respiratory Syndrome Coronavirus 2: A Prospective Study of Primary Infections and Immunizations
Source: J Infect Dis. 2026 Mar 5;233(6):e1374–85. doi: 10.1093/infdis/jiag068 (PMC13271396; doi:10.1093/infdis/jiag068)
Supplement: jiag068_Supplementary_Data [file jiag068_supplementary_data.pdf]

Anti-spike IgG avidity enhances neutralisation of SARS-CoV-2: a prospective study of primary infections and immunisations

Visa Nurmi, Lea Hedman, Katariina Vapalahti, Jussi Hepojoki, Hasan Uğurlu, Rommel Iheozor-Ejiofor, Chanice Knight, Kalle Saksela, Anu Kantele, Klaus Hedman, and Olli Vapalahti

Supplementary materials:

Supplementary methods

Anti-spike and anti-nucleoprotein IgG, IgA and IgM assays

Statistical analyses

Mixed multivariable models

Linear regression models

Receiver operating characteristic analyses

Supplementary tables

Supplementary table 1. The interaction model for Wuhan (B.1) neutralisation.

Supplementary table 2. The interaction model for Delta (B.1.617.2) neutralisation.

Supplementary table 3. Comparison of effects (slopes) in the interaction models.

Supplementary figures

Supplementary figure 1. Effect of IgG concentration on IgG avidity.

Supplementary figure 2. SARS-CoV-2 Beta variant (B.1.351) neutralisation titres

Supplementary figure 3. Regression lines depicting average kinetics of serological markers.

Supplementary figure 4. SARS-CoV-2 Wuhan to Beta and Wuhan to Delta neutralisation ratios

Supplementary figure 5. Effect of anti-spike IgM, IgG and IgG avidity on neutralisation titre

Supplementary figure 6. Correlation between measured and predicted neutralisation titre.

Supplementary figure 7. Wuhan neutralisation to anti-spike IgG ratio *vs.* anti-spike IgG avidity.

Supplementary figure 8. Diagnostic plots for regression model between Wuhan neutralisation to anti-spike IgG titre ratio and anti-spike IgG avidity.

Supplementary figure 9. Diagnostic plots for regression models between Wuhan to Beta and Wuhan to Delta neutralisation ratios and days after onset or first vaccine dose.

Supplementary figure 10. Diagnostic plots for regression model between relative anti-spike IgG avidity and relative anti-spike IgG level.

Supplementary methods

Anti-spike and anti-nucleoprotein IgG, IgA and IgM assays

Laboratory-derived anti-Wuhan spike (S) IgG, IgA and IgM and anti-Wuhan nucleoprotein (N) IgG ELISAs were used as described.[1,2]

End-point titres were acquired via three serial dilutions made at fourfold steps (e.g. 1:100, 1:400 and 1:1600). The working-dilution range of each sample was determined based on initial screening at 1:50 dilution, to include the titration end-point (i.e. high-titre samples were diluted more than those with low antiviral titres). Titration curves were generated as described[3] with the model $log(Absorbance) = B \times log(Dilution\ factor) + A$, fitted using the least squares method (A and B, fitting parameters). The titres (presented as arbitrary units, AU) were normalized against calibrator sera, which were assayed at each run, and which determined the titration end-point (i.e. the absorbance level at which the titres were acquired; e.g. for S-IgG and N-IgG, the end-point was the absorbance of the calibrator at 1:12800 dilution). Cutoffs for seropositivity were defined as average plus four standard deviations of sera collected prior to 2019, and were 69 AU/ml for S-IgG, 25 AU/ml for S-IgA, 39 AU/ml for S-IgM and 50 AU/ml for N-IgG.

Statistical analyses

Mixed multivariable models

Factors in statistical analyses were sex at birth, age, time after symptoms onset or first vaccine dose, group (hospitalised, non-hospitalised or vaccinee), and serological markers measured in the present study (Figure 1). Correlations were counted between all factors in each group and in the whole study population with R Hmisc package[4] and plotted using R corrplot package.[5] The mixed model (SAS Proc mixed procedure) was chosen as the most applicable method for modelling the clustered, longitudinal, and unbalanced (by time points) data. To consider the serial sampling of an individual, we used repeated statement of model with the compound symmetry (cs option of the statement) to specify the covariance structures for measurements of serial samples. Individuals were assigned as blocks for repeated measures with subject option.

Initial data of 14 hospitalised and 14 non-hospitalised patients, and 20 vaccinees comprised 53, 51 and 100 serum or plasma samples, respectively. The following were omitted from mixed models: one vaccinee with pre-vaccine SARS-CoV-2 infection, as the infection date was unknown; vaccinee samples collected before complete immunisation (2nd dose) and patient samples collected after booster immunisation, as antibody kinetics were not comparable with the other samples; 6 samples with missing values after interpolation (SAS proc expand SAS 9.4, SAS Institute, Cary NC, 2016); and one

hospitalised patient, which appeared to influence the results very strongly during the analysis. Consequently, the data analysed comprised 13 hospitalised and 14 non-hospitalised patients, and 19 vaccinees with 39, 45 and 57 samples, respectively, collected at 2 to 6 time points between 10 to 500 days after onset of symptoms or first vaccination. To balance the data, all factors were Log2-transformed and scaled between zero and one.

In multivariable mixed models predicting nAb titre (Wuhan or Delta), interactions to second order were examined and factors with a p-value less than 0.05 were considered to have a significant effect. Akaike information criterion (AIC; smaller is better), intraclass correlation coefficient (ICC; higher is better), normality of the residual distribution and linearity between the nAb titre and its predicted value were used to evaluate goodness of model fit. For groupwise comparisons, significance of differences of slopes (estimates) between neutralising antibodies and between groups were tested with Z-test.[6] Required homogeneity of group variances was tested with SAS proc glimmix using the covtest and homogeneity options in the model (SAS 9.4, SAS Institute, Cary NC, 2016). The Chi-square $p<0.05$ of the obtained covariance test indicates homogeneity of variance between groups. The small sample sizes did not allow corrections for multiple comparisons in the multivariable models and the pairwise correlations.

Linear regression models

Linear regression models were created with R 4.3.2 software (R Foundation for Statistical Computing, Vienna, Austria) and p-values of multiple comparisons were adjusted with Bonferroni-correction.

Samples with high (≥160) S-IgM titre were removed from regression model between log-transformed ratio of nAb-titre to S-IgG titre and log-transformed S-IgG avidity (Figure 2D). This was done to minimize confounding effect of IgM, as S-IgM has been shown to potently neutralise SARS-CoV-2[7]. Samples with low (<160) S-IgM titre were retained as they also comprised most low-avidity samples in the data, essential for observing full avidity maturation. For comparison, the regression model was applied to all samples, regardless of S-IgM titre (Supplementary figure 7). Diagnostic plots for the regression models are presented in Supplementary figure 8.

Regression models were created between log-transformed Wuhan (B.1) to Beta (B.1.351) and Wuhan to Delta (B.1.617.2) neutralisation titre ratios, and days after onset (patients) or first vaccine dose (vaccinees; Supplementary figure 4). Data-points with nAb-titre below (<20) or above (>20480) detection limit were omitted, as accurate ratios could not be calculated. The analysis included 105 samples with Beta variant and 135 samples with Delta variant. Diagnostic plots for the regression models are presented in Supplementary figure 9.

Confounding effect of S-IgG and N-IgG titre on respective IgG avidities was investigated by assaying each sample (n=140), for S and N-avidity, at three sets of dilutions, four-fold apart from each other (set-1, e.g. 1:100 - 1:1600, 16× relative S-IgG level; set-2, e.g. 1:400 - 1:6400, 4× relative S-IgG level; set-3, e.g. 1:1600 - 1:25600, 1× relative S-IgG level; Figure 2A-B, Supplementary figure 1). S and N avidity-results were calculated for each of the three dilution-sets (i.e. at three different IgG concentrations) with the LaviD[3] and *avidity index*[8] approaches. Data points with absorbance-values outside of linear range of the S-IgG and N-IgG assays, respectively, were omitted. S-avidity was investigated in more detail. Relative within-sample change in S-IgG avidity was calculated, with dilution set-2 as reference for the normalisation:

$$relative\ S\text{-}avidity\ at\ 16 \times S\text{-}IgG = (S\text{-}avidity, set\text{-}1)/(S\text{-}avidity, set\text{-}2)$$

$$relative\ S\text{-}avidity\ at\ 4 \times S\text{-}IgG = 1\ (reference)$$

$$relative\ S\text{-}avidity\ at\ 1 \times S\text{-}IgG = (S\text{-}avidity, set\text{-}3)/(S\text{-}avidity, set\text{-}2).$$

Regression model was then created between relative S-avidity and relative S-IgG level (Figure 2A-B). N.B. the reference point (relative S-avidity 1; relative S-IgG level 4×) was not included in the model as it already defined the other data points through the normalisation. Diagnostic plots for the regression models are presented in Supplementary figure 10.

Receiver operating characteristic analyses

Performance of S-IgG (n=103) and N-IgG (n=94) avidity in discriminating samples based on time after symptoms onset was investigated by receiver operating characteristic (ROC) analysis (Figure 2C). Area under curve (AUC) values were calculated, with R 4.3.2 software, for each sampling time point (e.g. AUC for discriminating samples collected within 60 days after onset from those collected later). N-IgG negative follow-up samples were omitted from the N-analysis (as seronegative samples do not have avidity-results either). The analysis included 103 samples with S-avidity and 94 samples with N-avidity.

References

1. Rusanen Juuso, Kareinen Lauri, Levanov Lev, Mero Sointu, Pakkanen Sari H., Kantele Anu, et al. A 10-Minute “Mix and Read” Antibody Assay for SARS-CoV-2. *Viruses* 2021;13(2). Doi: 10.3390/V13020143.
2. Stadlbauer Daniel, Amanat Fatima, Chromikova Veronika, Jiang Kaijun, Strohmeier Shirin, Arunkumar Guha Asthagiri, et al. SARS-CoV-2 Seroconversion in Humans: A Detailed Protocol for a Serological Assay, Antigen Production, and Test Setup. *Curr Protoc Microbiol* 2020;57(1). Doi: 10.1002/CPMC.100.
3. Nurmi Visa, Hedman Lea, Perdomo Maria F., Weseslindtner Lukas, Hedman Klaus. Comparison of approaches for IgG avidity calculation and a new highly sensitive and specific method with broad dynamic range. *Int J Infect Dis* 2021;110:479–87. Doi: 10.1016/J.IJID.2021.05.047.
4. Harrell Frank E Jr. Hmisc: Harrell Miscellaneous. R package version 4.6-0. *CRAN: Contributed Packages* 2021. Doi: 10.32614/CRAN.PACKAGE.HMISC.
5. Wei Taiyun, Simko Viliam. R package “corrplot”: Visualization of a Correlation Matrix (Version 0.92). *CRAN: Contributed Packages* 2021. Doi: 10.32614/CRAN.PACKAGE.CORRPLOT.
6. Paternoster Raymond, Brame Robert, Mazerolle Paul, Piquero Alex. USING THE CORRECT STATISTICAL TEST FOR THE EQUALITY OF REGRESSION COEFFICIENTS. *Criminology* 1998;36(4):859–66. Doi: 10.1111/J.1745-9125.1998.TB01268.X.
7. Klingler Jéromine, Weiss Svenja, Itri Vincenza, Liu Xiaomei, Oguntuyo Kasopefoluwa Y., Stevens Christian, et al. Role of Immunoglobulin M and A Antibodies in the Neutralization of Severe Acute Respiratory Syndrome Coronavirus 2. *J Infect Dis* 2020;223(6):957. Doi: 10.1093/INFDIS/JIAA784.
8. Hedman Klaus, Seppälä Ilkka. Recent rubella virus infection indicated by a low avidity of specific IgG. *J Clin Immunol* 1988;8(3):214–21. Doi: 10.1007/BF00917569.

Supplementary tables

Supplementary table 1. The interaction model for Wuhan (B.1) neutralisation.

Effect estimates of the mixed multivariable model best predicting Wuhan neutralisation titre. The model included anti-Wuhan serological markers S-IgG, S-IgM and S-IgG avidity, confounding factors sex, age, group (hospitalised, non-hospitalised or vaccine), days since onset of symptoms or since first vaccination (days), interaction of sex with days, and interactions of group with S-IgG, S-IgM and S-IgG avidity. Negative estimate value means inverse correlation, e.g. neutralisation titre degreases as days after onset of symptoms or first vaccination (days) increase, and vice versa. CI = 95 % confidence interval; * = p < 0.05.

Effect	Sex	Group	Estimate	Standard error	p	Lower CI	Upper CI
**Days**	M	-	-0.527	0.086	<0.0001*	-0.697	-0.357
	F	-	-0.352	0.074	<0.0001*	-0.498	-0.205
**Sex**	M	-	0.501	0.024	<0.0001*	0.453	0.550
	F	-	0.506	0.018	<0.0001*	0.469	0.543
**Age**	-	-	-0.148	0.050	0.005*	-0.249	-0.046
**Group**	-	Non-hospitalised	0.400	0.036	<0.0001*	0.327	0.473
	-	Hospitalised	0.695	0.027	<0.0001*	0.641	0.750
	-	Vaccinees	0.415	0.023	<0.0001*	0.369	0.461
**S-IgG**	-	Non-hospitalised	0.336	0.130	0.012*	0.076	0.595
	-	Hospitalised	0.203	0.120	0.095	-0.036	0.441
	-	Vaccinees	0.758	0.086	<0.0001*	0.588	0.929
**S-IgM**	-	Non-hospitalised	0.347	0.170	0.044*	0.009	0.684
	-	Hospitalised	0.148	0.086	0.090	-0.023	0.319
	-	Vaccinees	-0.178	0.092	0.055	-0.360	0.004
**S-IgG avidity**	-	Non-hospitalised	0.319		0.001*	0.139	0.498
	-	Hospitalised	0.108	0.098	0.277	-0.088	0.304
	-	Vaccinees	0.283	0.123	0.024*	0.038	0.528

**Model evaluation criterion**	**Criterion value**
Akaike information criterion (the smaller the better)	-260.1
Intraclass correlation coefficient (the higher the better)	0.534
Homoscedasticity p-value	0.730
Measured vs. predicted neutralisation titre, adjusted R^2	0.865

Supplementary table 2. The interaction model for Delta (B.1.617.2) neutralisation.

Effect estimates of a mixed multivariable model predicting Delta neutralisation titre. For comparison, the same model was used as with Wuhan neutralisation, and it included anti-Wuhan serological markers S-IgG, S-IgM and S-IgG avidity, confounding factors sex, age, group (hospitalised, non-hospitalised or vaccine), days since onset of symptoms or since first vaccination (days), interaction of sex with days, and interactions of group with S-IgG, S-IgM and S-IgG avidity. Negative estimate value means inverse correlation, e.g. neutralisation titre degreases as days after onset of symptoms or first vaccination (days) increase, and vice versa. CI = 95 % confidence interval; * = p < 0.05.

Effect	Sex	Group	Estimate	Standard error	p	Lower CI	Upper CI
**Days**	M	-	-0.390	0.097	<0.0001*	-0.583	-0.198
	F	-	-0.248	0.082	0.004*	-0.412	-0.084
**Sex**	M	-	0.344	0.024	<0.0001*	0.295	0.392
	F	-	0.339	0.019	<0.0001*	0.302	0.377
**Age**	-	-	-0.044	0.049	0.384	-0.143	0.056
**Group**	-	Non-hospitalised	0.268	0.038	<0.0001*	0.192	0.345
	-	Hospitalised	0.495	0.027	<0.0001*	0.440	0.550
	-	Vaccinees	0.261	0.023	<0.0001*	0.215	0.307
**S-IgG**	-	Non-hospitalised	0.304	0.140	0.033*	0.025	0.583
	-	Hospitalised	-0.075	0.135	0.579	-0.343	0.193
	-	Vaccinees	0.678	0.093	<0.0001*	0.494	0.862
**S-IgM**	-	Non-hospitalised	0.338	0.177	0.060	-0.014	0.690
	-	Hospitalised	0.192	0.096	0.049*	0.001	0.383
	-	Vaccinated	-0.211	0.102	0.041*	-0.413	-0.009
**S-IgG avidity**	-	Non-hospitalised	0.319	0.100	0.002*	0.121	0.518
	-	Hospitalised	-0.077	0.109	0.483	-0.294	0.140
	-	Vaccinees	0.191	0.136	0.164	-0.080	0.462

**Model evaluation criterion**	**Criterion value**
Akaike information criterion (the smaller the better)	-235
Intraclass correlation coefficient (the higher the better)	0.413
Homoscedasticity p-value	0.047*
Measured vs. predicted neutralisation titre, adjusted R^2	0.807

Supplementary table 3. Comparison of effects (slopes) in the interaction models. Multivariable mixed models including interactions between group and serological markers with Wuhan (B.1) or Delta (B.1.617.2) neutralising antibody titre as dependent variable. * = p < 0.05

Comparison between 1	and 2	Group	Variant	Effect 1	Effect 2	Standard error 1	Standard error 2	Z-value	p
**S-IgG titre**									
Wuhan	Delta	Non-hospitalised	-	0.336	0.304	0.012	0.013	0.207	0.836
Wuhan	Delta	Hospitalised	-	0.203	-0.075	0.011	0.012	1.858	0.063
Wuhan	Delta	Vaccinees	-	0.758	0.678	0.010	0.011	0.566	0.572
Wuhan	Delta	All	-	0.476	0.340	0.011	0.013	0.878	0.380
Non-hospitalised	Hospitalised	-	Wuhan	0.336	0.203	0.012	0.011	0.893	0.372
Non-hospitalised	Vaccinees	-	Wuhan	0.336	0.758	0.012	0.010	2.900	0.004*
Hospitalised	Vaccinees	-	Wuhan	0.203	0.758	0.011	0.010	3.891	<0.001*
Non-hospitalised	Hospitalised	-	Delta	0.304	-0.075	0.013	0.012	2.431	0.015*
Non-hospitalised	Vaccinees	-	Delta	0.304	0.678	0.013	0.011	2.462	0.014*
Hospitalised	Vaccinees	-	Delta	-0.075	0.678	0.012	0.011	5.047	<0.001*
**S-IgM titre**									
Wuhan	Delta	Non-hospitalised	-	0.347	0.338	0.013	0.014	0.053	0.957
Wuhan	Delta	Hospitalised	-	0.148	0.192	0.010	0.011	0.304	0.761
Wuhan	Delta	Vaccinees	-	-0.178	-0.211	0.010	0.011	0.230	0.818
Wuhan	Delta	All	-	0.076	0.125	0.011	0.013	0.318	0.751
Non-hospitalised	Hospitalised	-	Wuhan	0.347	0.148	0.013	0.010	1.319	0.187
Non-hospitalised	Vaccinees	-	Wuhan	0.347	-0.178	0.013	0.010	3.482	0.000*
Hospitalised	Vaccinees	-	Wuhan	0.148	-0.178	0.010	0.010	2.304	0.021*
Non-hospitalised	Hospitalised	-	Delta	0.338	0.192	0.014	0.011	0.931	0.352
Non-hospitalised	Vaccinees	-	Delta	0.338	-0.211	0.014	0.011	3.496	<0.001*
Hospitalised	Vaccinees	-	Delta	0.192	-0.211	0.011	0.011	2.731	0.006*
**S-IgG avidity**									
Wuhan	Delta	Non-hospitalised	-	0.319	0.319	0.010	0.011	0.004	0.997
Wuhan	Delta	Hospitalised	-	0.108	-0.077	0.010	0.011	1.256	0.209
Wuhan	Delta	Vaccinees	-	0.283	0.191	0.011	0.012	0.613	0.540
Wuhan	Delta	All	-	0.271	0.175	0.011	0.014	0.613	0.540
Non-hospitalised	Hospitalised	-	Wuhan	0.319	0.108	0.010	0.010	1.461	0.144
Non-hospitalised	Vaccinees	-	Wuhan	0.319	0.283	0.010	0.011	0.249	0.803
Hospitalised	Vaccinees	-	Wuhan	0.108	0.283	0.010	0.011	1.206	0.228
Non-hospitalised	Hospitalised	-	Delta	0.319	-0.077	0.011	0.011	2.630	0.009*
Non-hospitalised	Vaccinees	-	Delta	0.319	0.191	0.011	0.012	0.844	0.399
Hospitalised	Vaccinees	-	Delta	-0.077	0.191	0.011	0.012	1.772	0.076

Supplementary figures

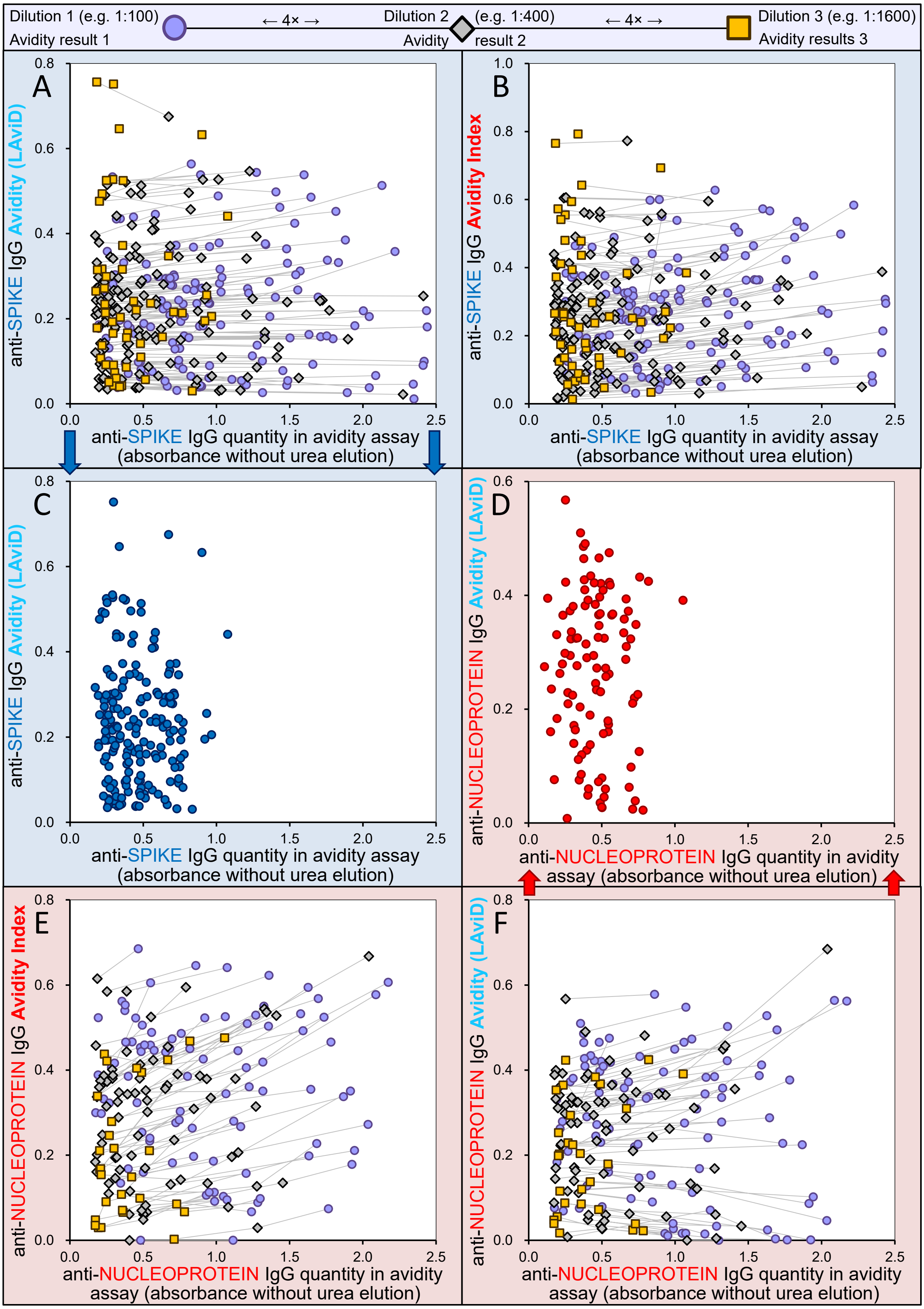

Supplementary figure 1. Effect of anti-Wuhan spike (S; **A-C**) and anti-Wuhan nucleoprotein (N; **D-E**) IgG concentration on S-IgG and N-IgG avidities within linear range of the present avidity assay. Each sample (n=140) was assayed at three sets of dilutions, four-fold apart from each other. From the same raw data, avidities were obtained with the LaviD-method (**A, C, D, F**; Ref. Nurmi et al. IJID 2021) used in the present study and, for comparison, with the popular "avidity index" approach (**B, E**; Ref. Hedman & Seppälä JCI 1988). For acquisition of final avidity results, the S-IgG and N-IgG quantities were normalized (**C, D**) by selecting the working dilution of each sample based on the initial screening.

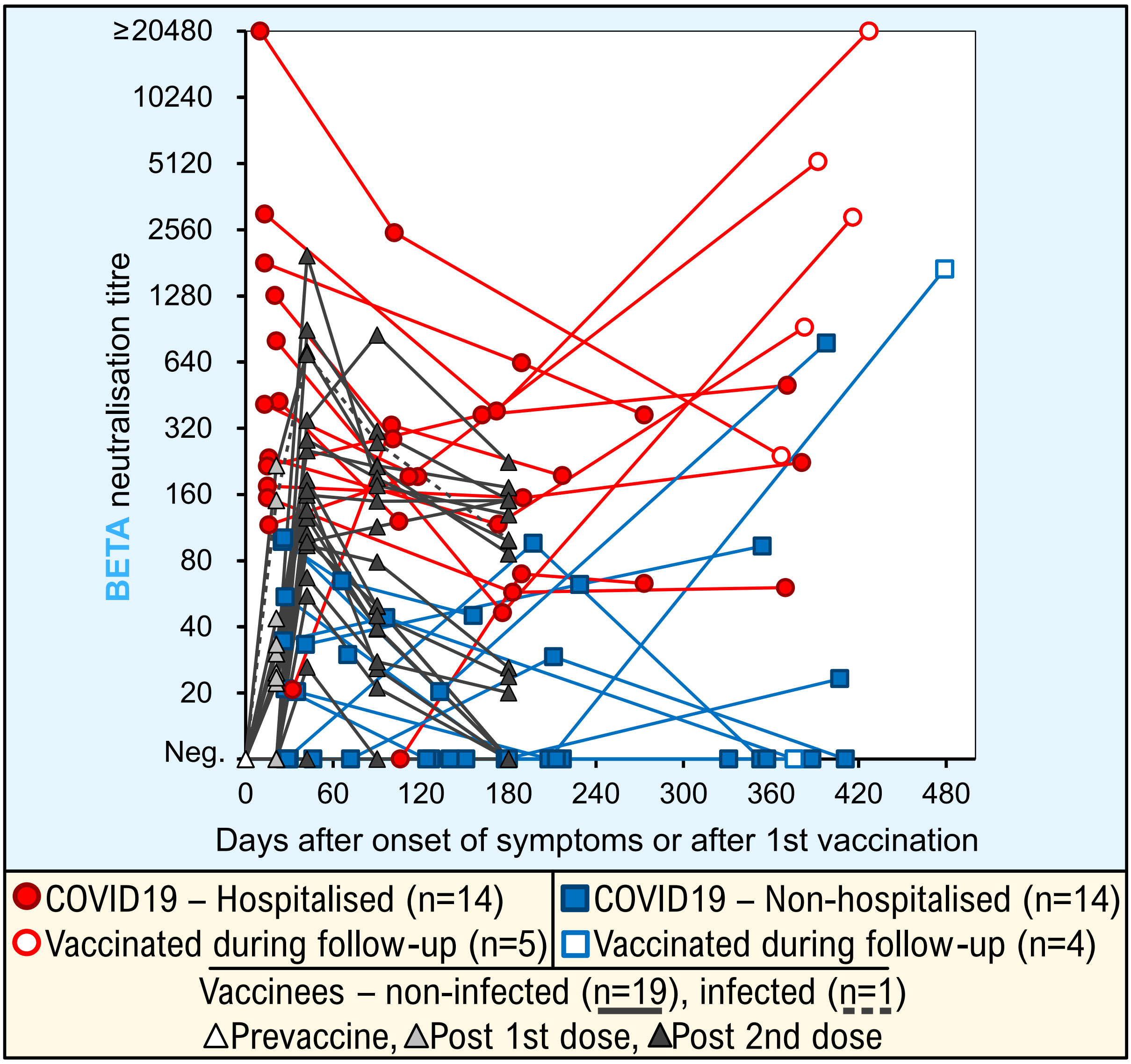

Supplementary figure 2. SARS-CoV-2 Beta variant (B.1.351) neutralisation titres of hospitalised and non-hospitalised COVID-19 patients and vaccinees in relation to time after onset (patients) or first vaccine dose (vaccinees).

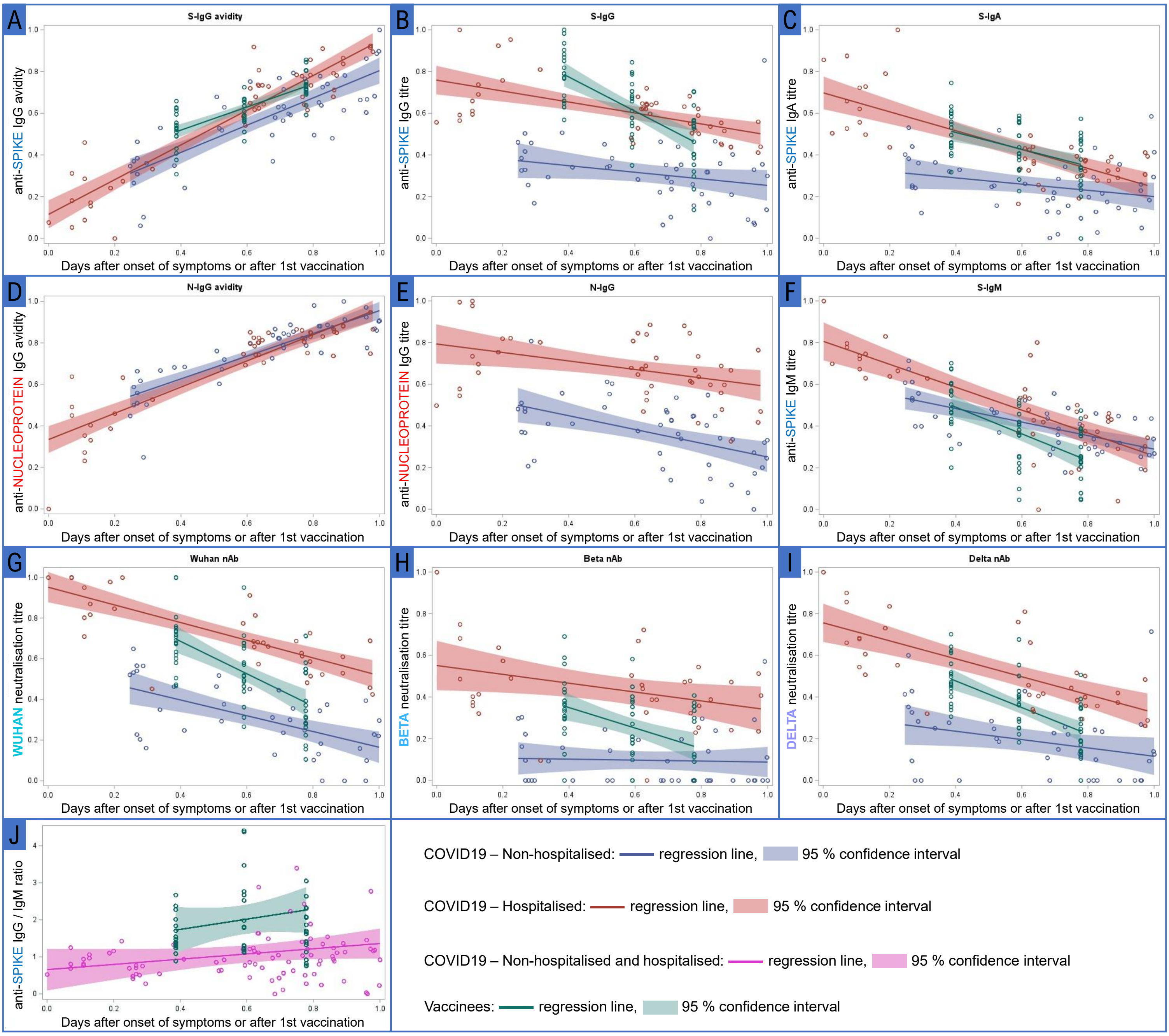

Supplementary figure 3. Regression lines depicting average kinetics of serological markers. Anti-Wuhan spike (S) IgG avidity (**A**), S-IgG titres (**B**), S-IgA titres (**C**), anti-Wuhan nucleoprotein (N) IgG avidity (**D**), N-IgG titres (**E**), S-IgM titres (**F**), Wuhan (B.1) neutralisation titres (**G**), Beta (B.1.351) neutralisation titres (**H**) and Delta (B.1.617.2) neutralisation titres (**I**) of hospitalised and non-hospitalised COVID-19 patients and vaccinees in relation to time after onset (patients) or first vaccine dose (vaccinees). All factors were Log2-transformed and scaled between zero and one. S-IgG to S-IgM ratio was calculated using the scaled values (**J**).

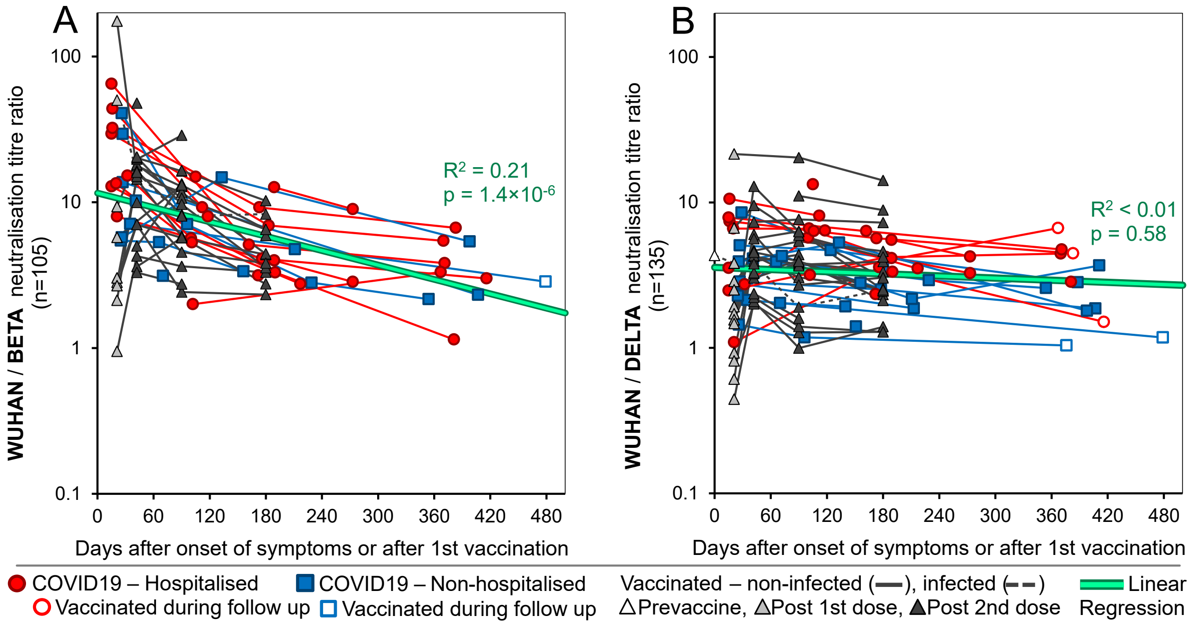

Supplementary figure 4. SARS-CoV-2 Wuhan (B.1) to Beta (B.1.351; A) and Wuhan to Delta (B.1.617.2; B) neutralisation titre ratios

of hospitalised and non-hospitalised COVID-19 patients and vaccinees in relation to time after onset (patients) or first vaccine dose (vaccinees). R^2, adjusted R-squared value. p, Bonferroni adjusted p-value.

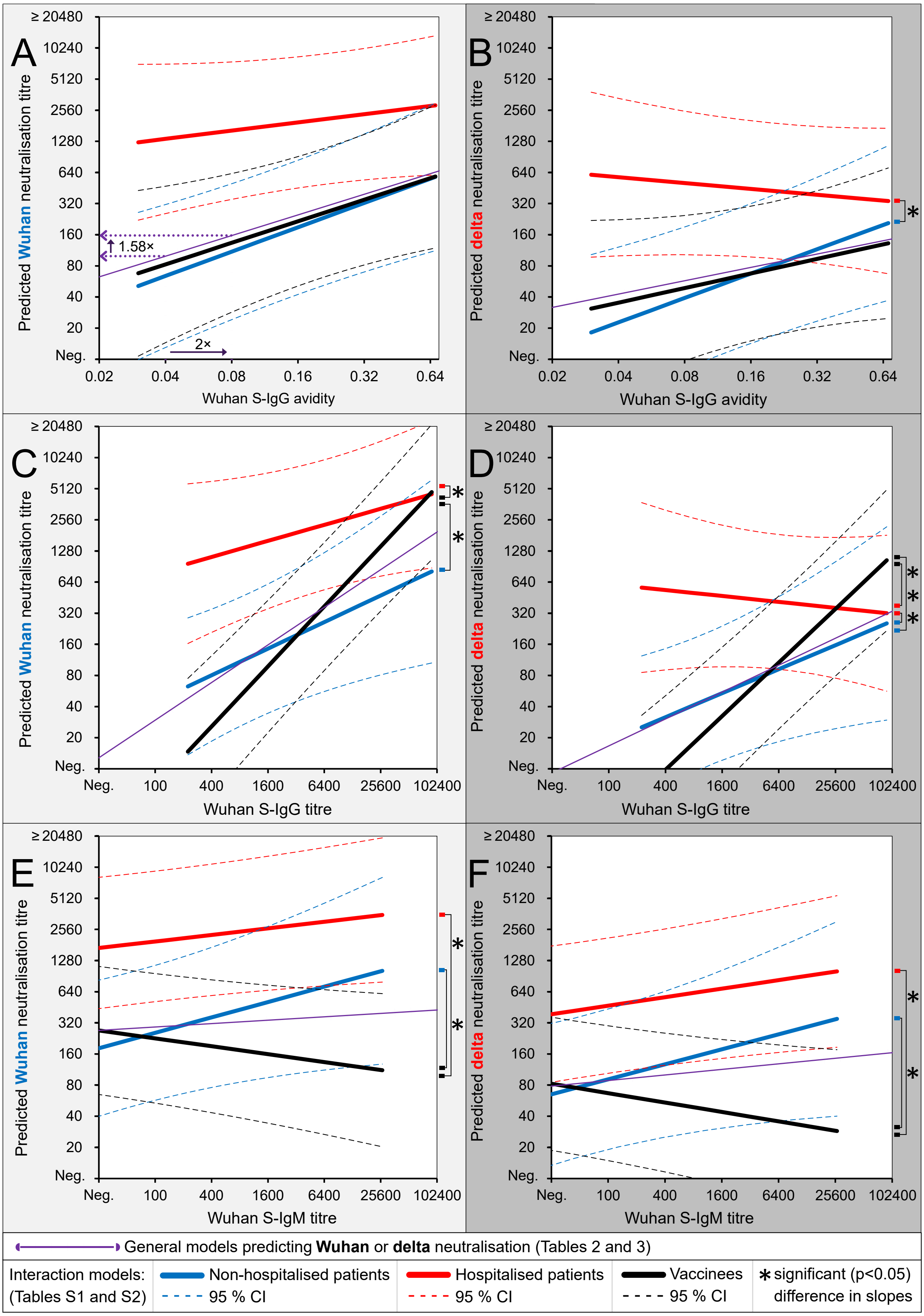

Supplementary figure 5. Effect of anti-Wuhan spike (S) IgG avidity (A; B), S-IgG titre (C; D) and S-IgM titre (E; F) on Wuhan (B.1) and Delta (B.1.617.2) neutralisation titres, respectively.

Graphs show predicted neutralisation in mixed multivariable models and at constant value of other variables, e.g. change in neutralisation when quantity of IgG remains same but their avidity increases.

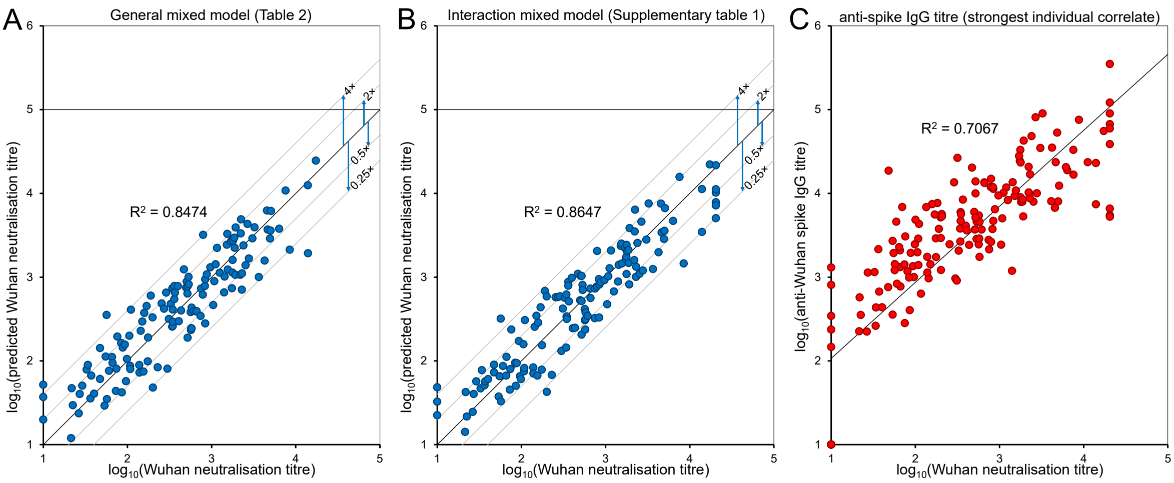

Supplementary figure 6. Correlation between measured and predicted Wuhan (B.1) neutralisation titre of hospitalised and non-hospitalised COVID-19 patients and vaccinees.

(A) Wuhan neutralisation titre predicted with the General mixed model (Table 2),

(B) Wuhan neutralisation titre predicted with the Interaction mixed model (Supplementary table 1),

(C) for comparison, anti-Wuhan spike IgG titre which was the strongest individual correlate of the measured neutralisation titre.

The light diagonal lines in panels A and B correspond to prediction accuracy, i.e. whether the predicted value is twice (or four-fold) as high, or half (or quarter) of the measured neutralisation titre. R^2, adjusted R-squared value.

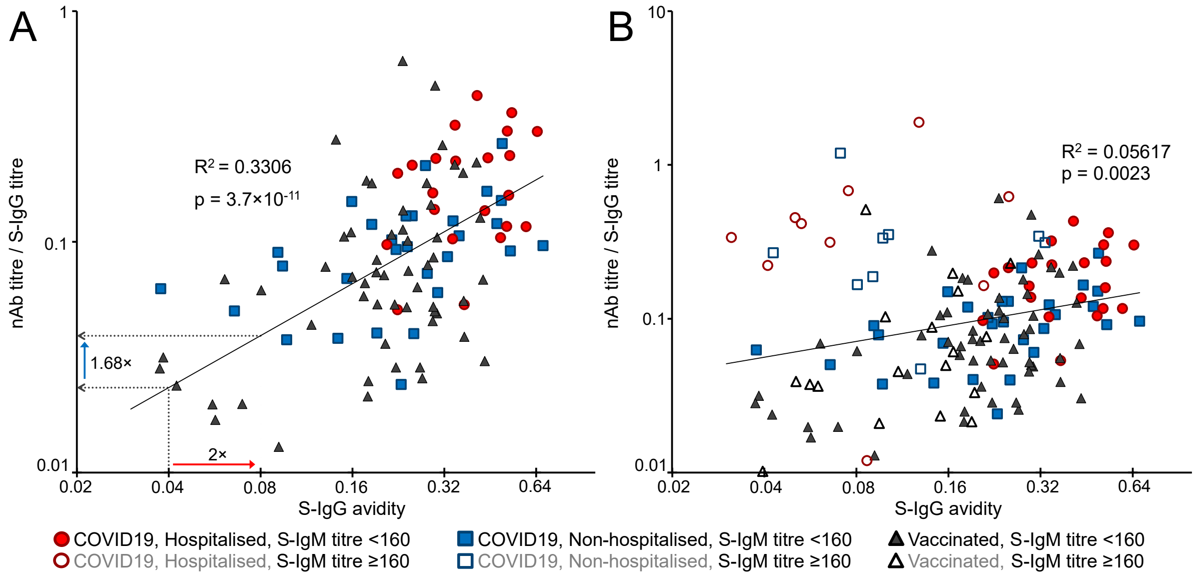

Supplementary figure 7. Wuhan (B.1) neutralising antibody (nAb) titre to Wuhan anti-spike (S) IgG titre ratio in relation to Wuhan S-IgG avidity.

S-IgM has been shown to potently neutralise SARS-CoV-2, and samples with high (≥160) S-IgM titre were omitted to minimize confounding effect of S-IgM (A). For comparison results are also shown for all samples regardless of S-IgM titre (B). Solid lines show linear regression between log-transformed nAb to S-IgG ratio and log-transformed S-IgG avidity. Based on the regression model in panel A, doubling of S-IgG avidity increased nAb to S-IgG ratio 1.68-fold, i.e. at constant S-IgG level the average neutralisation potency of an S-IgG molecule increased 1.68-fold. R^2, adjusted R-squared value. p, Bonferroni adjusted p-value.

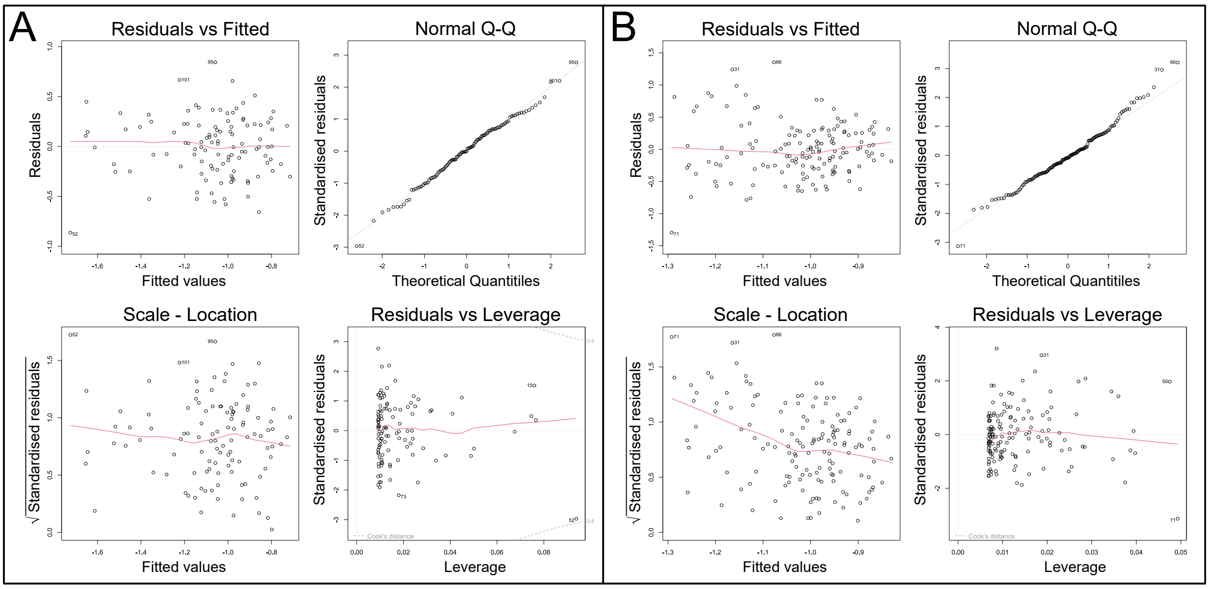

Supplementary figure 8. Diagnostic plots for linear regression models between log-transformed ratio of Wuhan (B.1) neutralising antibody titre to Wuhan anti-spike (S) IgG titre and log-transformed S-IgG avidity.

Samples with high (≥160) S-IgM titre were omitted to minimize confounding effect of S-IgM (**A**). For comparison, the regression model was applied to all samples, regardless of S-IgM titre (**B**).

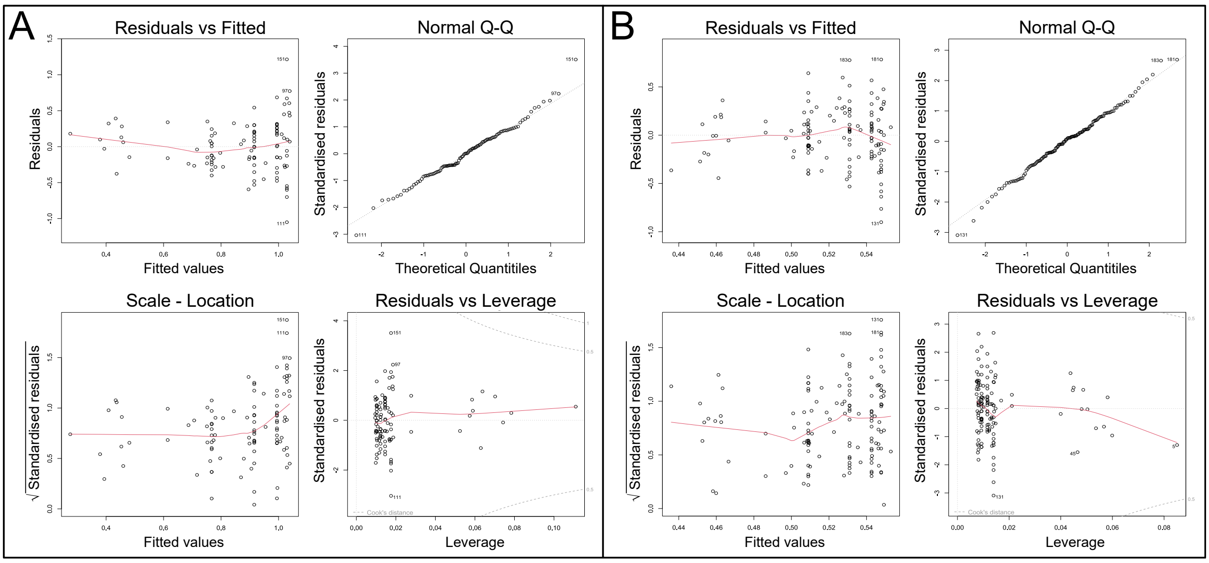

Supplementary figure 9. Diagnostic plots for linear regression models between log-transformed Wuhan (B.1) to Beta (B.1.351; **A**) and Wuhan to Delta (B.1.617.2; **B**) neutralisation titre ratios and days after onset (patients) or first vaccine dose (vaccinees).

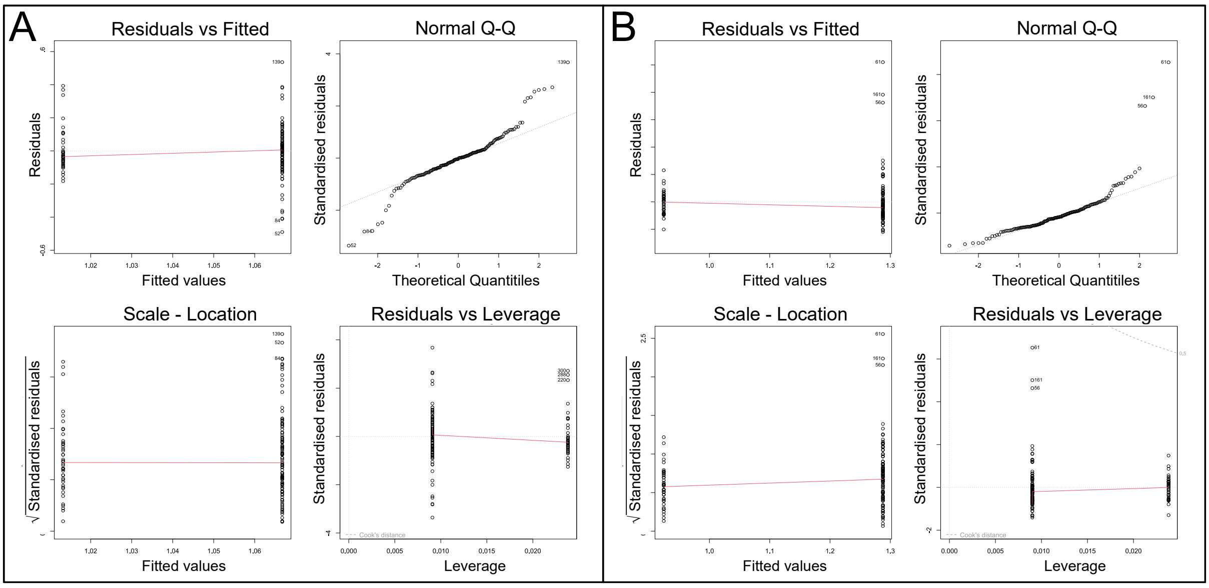

Supplementary figure 10. Diagnostic plots for linear regression models between relative anti-Wuhan spike (S) IgG avidity and relative S-IgG level.

Avidities were obtained with the LaviD-method (**A**; Ref. Nurmi et al. IJID 2021) used in the present study and, for comparison, with the popular “avidity index” approach (**B**; Ref. Hedman & Seppälä JCI 1988).
